# A versatile method for the UVA-induced cross-linking of acetophenone- or benzophenone-functionalized DNA

**DOI:** 10.1038/s41598-018-34892-9

**Published:** 2018-11-07

**Authors:** Jevgenija Jakubovska, Daiva Tauraitė, Rolandas Meškys

**Affiliations:** Department of Molecular Microbiology and Biotechnology, Institute of Biochemistry, Life Sciences Center, Vilnius University, Sauletekio al. 7, LT-10257 Vilnius, Lithuania

## Abstract

Bioconjugation, biosensing, bioimaging, bionanomaterials, etc., are only a few examples of application of functionalized DNA. Since base-modified nucleic acids contribute not only to a broad range of biotechnological fields but also to the understanding of various cellular processes, it is crucial to design novel modifications with unique properties. Here, we demonstrate the utilization of *N*^4^-cytidine modified oligonucleotides, which contain reactive acetophenone (AP) or benzophenone (BP) groups, for the UV-induced cross-linking. We find that terminal deoxynucleotidyl transferase-mediated 3′-tailing using AP/BP-containing modified nucleotides generates photoactive DNA, suitable for a straightforward covalent cross-linking with both interacting proteins and a variety of well-known solid polymeric supports. Moreover, we show that AP/BP-functionalization of nucleic acid molecules induces an efficient cross-linking upon exposure to UVA light. Our findings reveal that 3′-tailed single-stranded DNA bearing AP/BP-moieties is easily photoimmobilized onto untreated polystyrene, polypropylene, polylactate, polydimethylsiloxane, sol-gel and borosilicate glass substrates. Furthermore, we demonstrate that such immobilized DNA probes can be further used for successful hybridization of complementary DNA targets. Our results establish novel *N*^4^-cytosine nucleobase modifications as photoreactive labels and suggest an effortless approach for photoimmobilization of nucleic acids.

## Introduction

Bioconjugation of nucleic acids with diverse biomolecules as well as a variety of solid ligands is of great importance in medicine and bionanotechnology^1,2^. As such, oligonucleotide (ON) bioconjugates hold promise for targeted drug delivery^3,4^, bioimaging^5,6^, biosensing^7,8^, as well as for the development of DNA nanomaterials^9,10^ and DNA-based nanodevices^11,12^. Due to such a broad application range of DNA-based systems, a site-specific immobilization of DNA molecules and subsequent hybridization are the two major events to be developed and optimized. In addition, nucleic acid bioconjugation plays a significant role in elucidating many of cellular processes, mainly those involving protein-DNA/RNA interactions^13^. Thus, a precise knowledge of the contacts made by the transiently binding proteins and nucleic acid molecules is essential to unravel the mechanisms of protein-nucleic acid interaction as well as to understand the architecture of such assemblies.

The conjugation of nucleic acids to a ligand, whether it is a biomolecule or a solid support, is brought by the reaction of particular functional groups between the two targets. Many methods of nucleic acid labelling are available, which are generally based on linking a specific tag to a sugar phosphate backbone at either 3′- or 5′-end of a nucleic acid molecule. The ease and efficiency of DNA end functionalization using automated solid-phase synthesis dictates the choice of modification^14^. Meanwhile, the attachment of a particular group to the nucleobase is a much more attractive approach in terms of sequence-specific labelling and programmability. Apart from a greater repertoire of modifications, the base-alteration offers a discontinuous space for functionalization with highly reactive but rather sophisticated and unique substitutions, such as aldehyde, acrylamide, vinylsulfonamide, chloroacetamide, trifluoroacetophenone or click chemistry-based functional groups, that are suitable for various bioconjugation procedures^15–20^. Considering base-modified nucleotides, terminal deoxynucleotidyl transferase (TdT)-mediated 3′-end tailing is a very promising approach for the enzymatic preparation of functional DNA species^21–25^. In fact, a wide spectrum of base-modified DNA oligomers bearing reactive alkyne-, azide-, oxanine-, amine- or imidazole- moieties can be prepared during TdT-assisted tailing, and further applied for the bioconjugation with proteins, coupling with fluorophores or immobilization^26–29^.

Photochemical cross-linking is a powerful strategy for studying biomolecule interactions as well as for anchoring a variety of ligands on different surfaces. A general photo-induced covalent cross-linking procedure can be achieved either by using an external photolinker^30^ or by introducing a native photoactive functional group into a targeted molecule^31^, the latter being a more specific and efficient approach. Aryl ketones, aryl azides and diazirines are the most popular photoreactive groups used for the photo cross-linking^30,31^. In fact, these have been used to study the cross-linking of DNA-DNA^32^ or protein-DNA/RNA complexes^33–36^, to probe protein-protein interactions^37,38^ or to attach a variety of biomolecules to polymer surfaces^39,40^. In addition, novel click chemistry-based photo-crosslinkers (e.g., tetrazole) have proved to be very promising as alternative photo-labels^41^.

Benzophenone (BP) is the most widely used and versatile photophore in bioorganic chemistry and material science^42^. In addition to an extraordinary stability compared to other cross-linking agents, BP is activated in a 350–365 nm UV range, which avoids protein and nucleic acid damage. BP and its derivatives are widely used for the photo-cross-linking of polymers as well as for the modification of both two- and three-dimensional solid supports^42^. In addition, BP-induced photochemical immobilization, grafting and patterning enable generation of biocompatible materials^43–45^ or 3D scaffolds^46,47^. Such functionalized platforms can be further utilized for the construction of diverse molecular diagnostic tools, such as DNA microarrays^48,49^. Furthermore, BP-based photoprobes are very frequently used to map interactions between the two molecules^42^. It has been demonstrated that the interaction between proteins can be detected using a BP-containing amino acid that can be either genetically encoded^37^ or incorporated via solid-phase synthesis^50^. Similarly, protein-DNA complexes can be identified using a BP-bearing peptide^51^. Nevertheless, the most common approach to a BP-mediated photo-cross-linking is the application of BP as a discrete photoinitiator^52^. Regarding BP-induced photoimmobilization strategies, the solid support itself must be coated, chemically activated or otherwise pre-treated, thus limiting its utilization. It seems that despite a huge variety of BP-containing cross-linking agents, the time-consuming synthetic routes render its applications inflexible.

Recently, we have described a set of *N*^4^-modified 2′-deoxycytidine 5′-triphosphates, including acetophenone (AP) and BP moieties-bearing nucleotide analogues^53^. We have found that AP- and BP-containing cytidine nucleotides are efficiently incorporated by a variety of family A and B DNA polymerases. In addition, we have shown that TdT is able to generate single-stranded DNA (ssDNA) fragments bearing up to several hundred such functional groups. Here, we report on the application of AP- and BP-functionalized DNA for the UVA-induced cross-linking with interacting proteins and describe an AP- and BP-mediated DNA photoimmobilization on various untreated solid supports. The results may prove useful for both the development of a feasible and direct DNA photoimmobilization approach as well as for the discovery of novel DNA-protein interactions.

## Results

### UVA-induced photo-cross-linking of AP- or BP-bearing ONs to TdT

**Figure 1:**
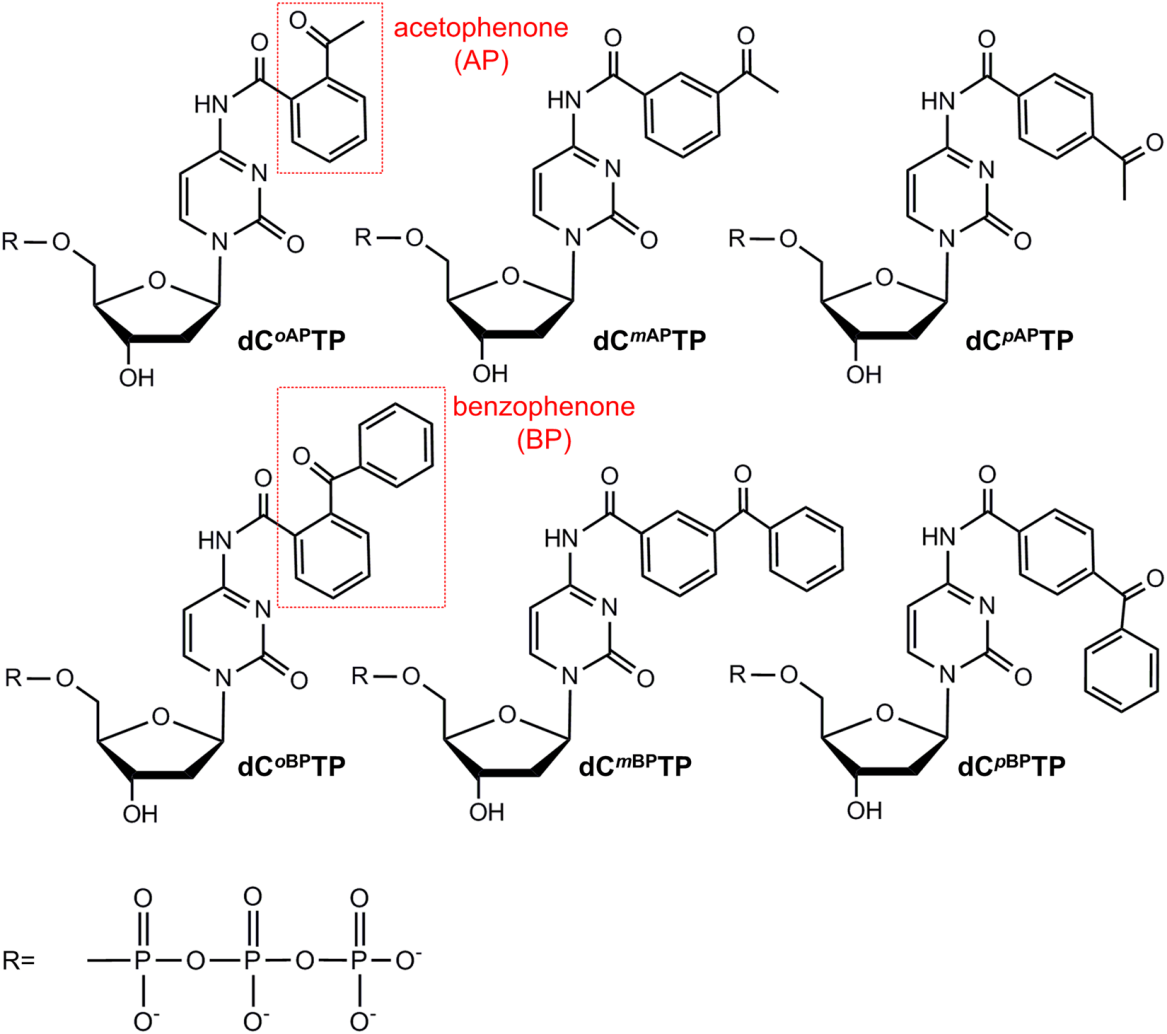
Chemical structures of *N*^4^-modified dCTPs used in this study. dC^*o*AP^TP: *N*^4^-(2-acetyl-benzoyl)-2′-deoxycytidine-5′-triphosphate, dC^*m*AP^TP: *N*^4^-(3-acetyl-benzoyl)-2′-deoxycytidine-5′-triphosphate, dC^*p*AP^TP: *N*^4^-(4-acetyl-benzoyl)-2′-deoxycytidine-5′-triphosphate; dC^*o*BP^TP: *N*^4^-(2-benzoyl-benzoyl)-2′-deoxycytidine-5′-triphosphate, dC^*m*BP^TP: *N*^4^-(3-benzoyl-benzoyl)-2′-deoxycytidine-5′-triphosphate, dC^*p*BP^TP: *N*^4^-(4-benzoyl-benzoyl)-2′-deoxycytidine-5′-triphosphate.

Our strategy for the synthesis of reactive ON photoprobes bearing AP- or BP-functional groups exploited the TdT-based template-independent 3′-elongation. A set of *N*^4^-modified cytidine triphosphates was used, comprising a series of AP- or BP-containing nucleotide isomers (Fig. 1). TdT-mediated 3′-end tailing using the aforementioned cytidine analogues was described previously^53^. To examine a feasibility of AP- or BP-induced covalent cross-linking of modified DNA with a DNA-binding protein, we have chosen TdT as a particularly relevant model for the 3′-end-specific DNA-binding. This enabled us to simplify the process and perform a photo-cross-linking procedure immediately after the completion of elongation reactions.

**Figure 2:**
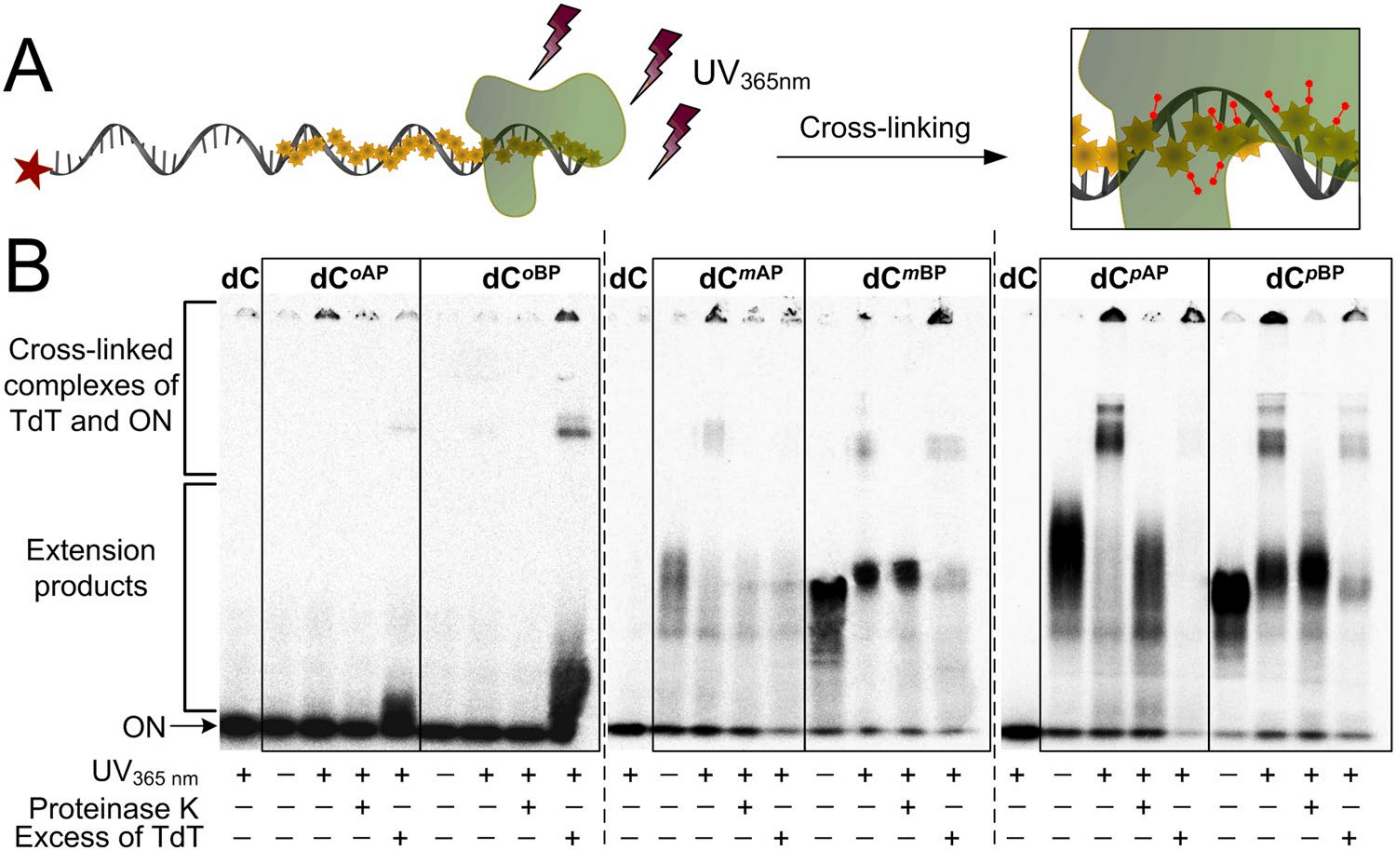
UVA-induced cross-linking of AP- or BP-containing DNA to TdT. (**A**) A scheme of the photo-cross-linking between 3′-modified DNA and DNA-binding protein upon exposure to UV_365 nm_ light. (**B**) Autoradiograms of SDS-PAGE showing covalent bioconjugates of the indicated 3′-modified ONs and TdT. The used nucleotides are indicated above the lanes.

Figure 2 shows the formation of covalent conjugates after exposure to a 365 nm UV light, where DNA-TdT cross-links are represented by the bands with lower mobility. Since a one-pot photochemical cross-linking approach was utilized, a mixture of DNA photoprobes of different length caused the formation of several conjugates, explaining the multiple bands present. In general, the yield of the cross-linking strongly depended on the number of photoreactive groups present on a DNA molecule. Since both *para*-isomers were revealed to be better substrates for TdT than *ortho*- or *meta*-analogues^53^, the cross-linking using *p*AP- or *p*BP-containing DNA was the most efficient (Fig. 2B). Moreover, the addition of an excessive amount of TdT led to an even more effective formation of covalent complexes. If the length of the modified DNA must be constant, or the amount of the targeted protein is limiting, one can prolong the time of irradiation rather than vary the critical parameters.

It was noticeable that after UV irradiation, a considerable part of material was unable to enter the gel, particularly in the case of *p*AB or *p*BB modifications (Fig. 2B). To determine its origin, the irradiated samples were further treated with protease. The disappearance of all the bands with the lower mobility (including the ones at the wells) demonstrated that all covalent adducts formed were DNA-protein rather than DNA-DNA conjugates (Fig. 2B). Due to a high number of photoreactive as well as highly hydrophobic groups present in a nucleic acid molecule, formation of multiple covalent bonds or specific nanostructured aggregates, or both, could occur.

To investigate the specificity of the cross-linking, several control reactions were done. Therefore, after the completion of elongation reactions, TdT was removed following the addition of other protein targets and subsequent exposure to UV radiation. To show that AP/BP modification-containing DNA can be cross-linked not only with TdT but with other DNA binding proteins as well, we attempted to couple modified ON with a single-stranded DNA binding protein (SSB) as a representative. Native-PAGE revealed formation of several dC^*p*BP^-ON:SSB cross-linked products that can be explained by the heterogeneity of the modified DNA as well as the multimericity of SSB (Supplementary Fig. S1). The requirement of a specific DNA-protein interaction for a successful cross-linking was also confirmed by the negative control experiment utilizing BSA where no covalent adducts were detected (Supplementary Fig. S1).

### Photoimmobilization of *N*^4^-modified DNA onto solid supports

Next, we performed a covalent attachment of ssDNA, via its modified 3′-*m*AP- or *m*BP-containing tail, to a variety of intact solid substrates. Polystyrene (PS), polypropylene (PP), polylactate (PLA), polydimethylsiloxane (PDMS), borosilicate (BS) glass and sol-gel based composites served as model polymer supports. Accordingly, the untreated ON as well as ssDNA oligomers 3′-extended using a natural dCTP were used as the negative control.

**Figure 3:**
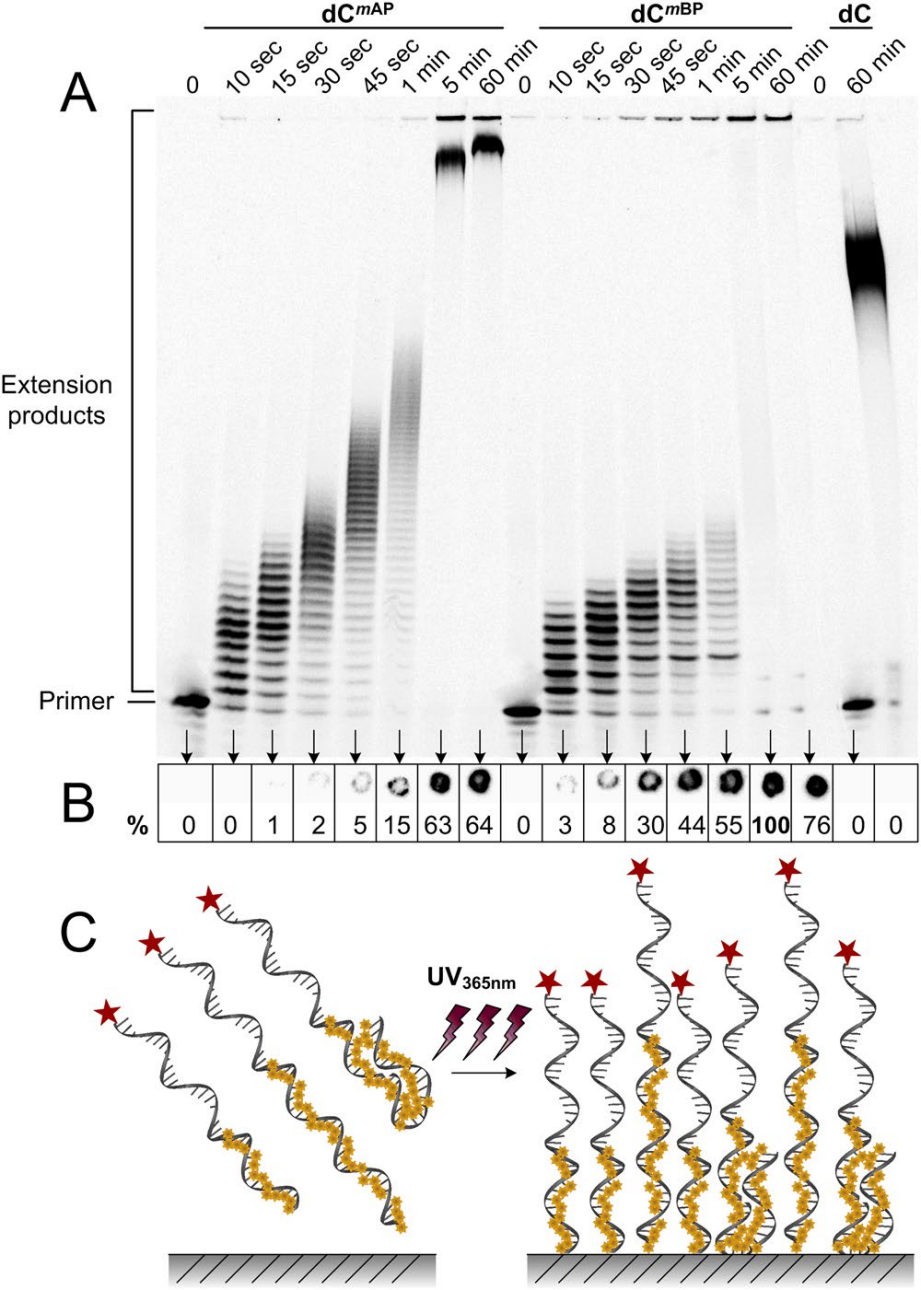
Photoimmobilization of AP- or BP-containing DNA to a PS support. (**A**) An autoradiogram of denaturing polyacrylamide gel showing TdT-catalysed 3′-end tailing with dCTP, dC^*m*AP^TP and dC^*m*BP^TP as substrates. The used nucleotides and elongation time are indicated above the lanes. (**B**) An autoradiogram of UVA-induced photoimmobilization of dC^*m*AP^-ONs and dC^*m*BP^-ONs to PS. The relative percentage of immobilization is indicated at the bottom where the most efficient immobilization was chosen to serve as 100%. Data represents the average of three independent experiments. (**C**) A schematic representation of AP- or BP-induced DNA photoimmobilization.

To investigate the dependence of the photoimmobilization efficiency on the number of photoreactive groups present in a nucleic acid molecule, the duration of the 3′-tailing reaction was varied. In fact, ranging the duration of TdT-mediated elongation from 10 sec to 60 min, several to several hundred of photoinducable nucleotides were appended, respectively (Fig. 3A). In the case of BP-modified DNA products, a considerable portion of such radioactive material was unable to enter the gel (Fig. 3A). Performing elongation reactions in the dark did not showed any changes (data not shown). Surprisingly, this bulky material was unable to enter the agarose gel also (Supplementary Fig. S2). In fact, to mobilize such large DNA, pulse-field gel electrophoresis was performed, yet we did not observe any migration of the modified DNA whatsoever (Supplementary Fig. S3).

The direct impact of the amount of AP- or BP-moieties on DNA attachment efficiency is illustrated in Fig. 3. Clearly, the more photoactive modifications (either AP or BP) that were present on an ON, the better such ON was attached to a PS substrate. The data presented in Fig. 3 indicate that a BP-functionalization induced a more effective immobilization. Indeed, only a few BP-modifications gave superior immobilization results compared to those observed for a large number of AP-groups. Neither untreated ON, nor 3′-dC-extended DNA fragments showed any indications of immobilization (Fig. 3).

**Figure 4:**
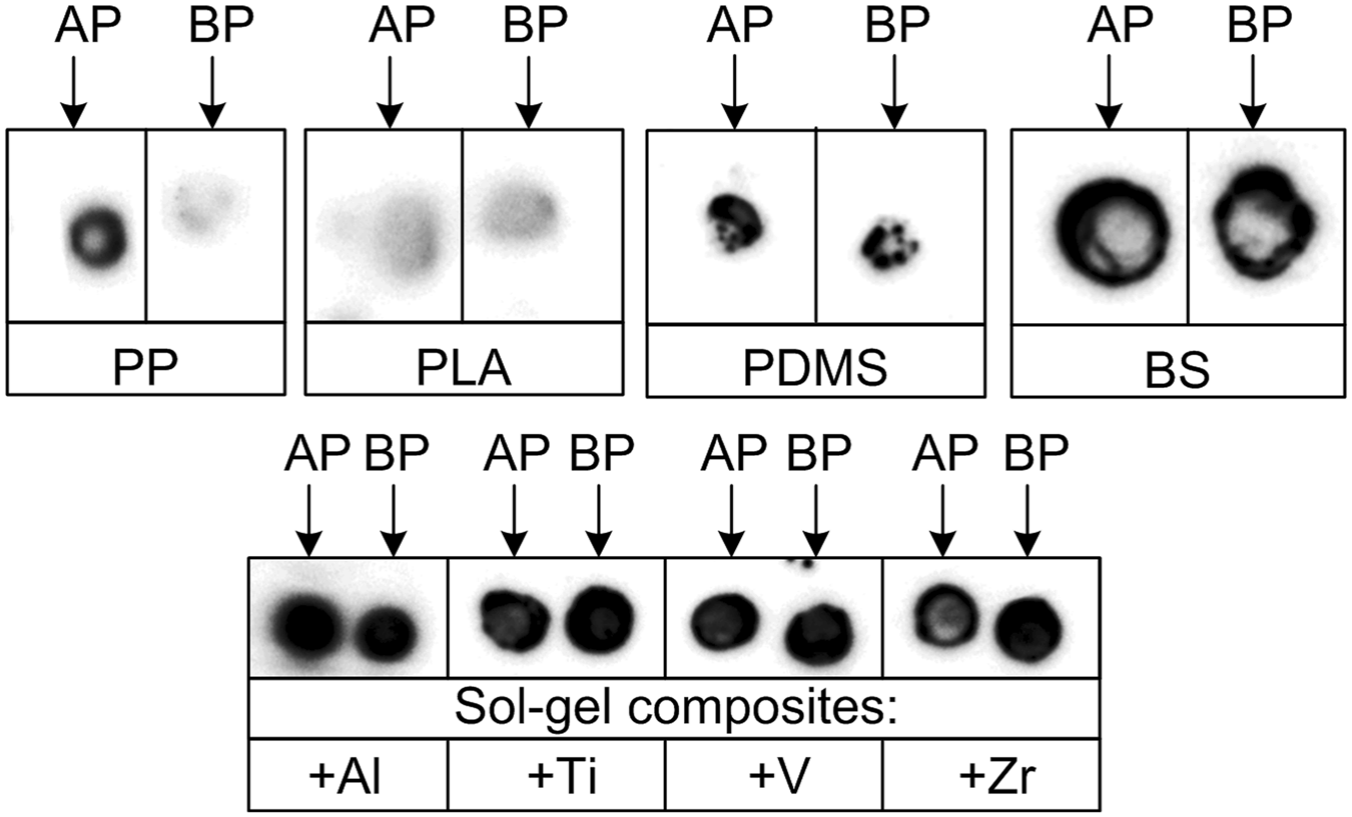
Autoradiograms of UVA-induced photoimmobilization of dC^*m*AP^-ONs and dC^*m*BP^-ONs to different solid supports. The used photoreactive probes and solid supports are indicated above and below each autoradiogram, respectively.

Further, a successful photoimmobilization of AP- or BP-modified DNA was accomplished using PP, PLA, PDMS, BS as substrates as well as metal-containing (Al, Ti, Zr or V) sol-gel based hybrid materials (Fig. 4). In general, both AP- and BP-containing DNA demonstrated similar UVA-induced immobilization efficiencies on every solid substrate tested.

### Hybridization of DNA targets to photoimmobilized BP-containing DNA probes

To verify that the attached 3′-dC^BP^-tailed DNA probes are properly oriented for a successful detection of complementary DNA, we performed a target–probe hybridization procedure. To detect only specific DNA sequences, two different ssDNA probes P1 and P2 were chosen. Both were elongated at the 3′-end using TdT and dC^*p*BP^TP, and photoimmobilized onto PS plates. A 5′-radiolabelled ssDNA fragment P1R served as a DNA target. P1R sequence perfectly matched the DNA probe P1, whilst the thermal stability of P1R-P2 dimer was extremely low. Therefore, a hybridization signal upon formation of P1-P1R dimer represented a positive detection system.

**Figure 5:**
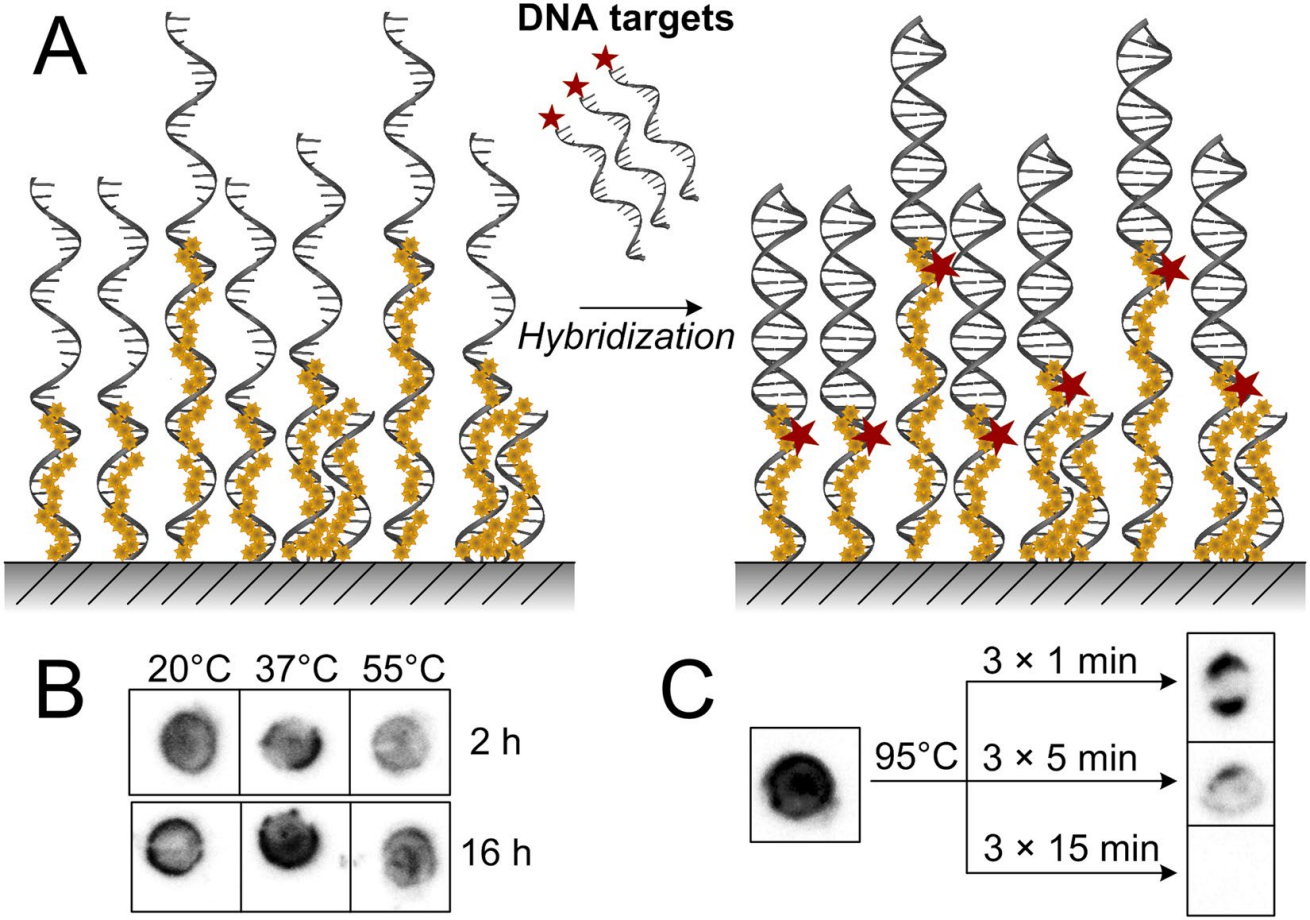
Hybridization of DNA targets to photoimmobilized 3′-dC^*p*BP^-DNA probes on a PS support. (**A**) A scheme visualizing hybridization experiment. (**B**) An autoradiogram of hybridization of 5′-radiolabelled ssDNA targets to photoimmobilized BP-bearing complementary DNA probes. (**C**) An autoradiogram of DNA denaturation of 5′-radiolabelled DNA targets. The used hybridization and denaturation conditions are indicated.

To determine targets with different sequence similarity, various hybridization conditions were tested. High stringency hybridization conditions (e.g., high temperature, low salt concentration) permit hybridization only between highly similar nucleic acid molecules, whereas low stringency conditions (low temperature, high salt) allow unspecific hybridization. Remarkably, our results demonstrated that even under low stringency hybridization conditions (20 °C, ~0.8 M salt), hybridization occurred specifically between complementary sequences (Fig. 5B). Moreover, the detection of complementary DNA targets was possible in a wide temperature range (20–55 °C) as well as within short time periods (2 hours).

Finally, to examine the suitability of 3′-dC^BP^-tailed DNA probes for the construction of DNA microarrays, we performed a facile procedure for DNA denaturation. To avoid harsh denaturation methods, such as alkaline or dimethyl sulfoxide treatment, we tested the most common and secure way to denature double-stranded-DNA (dsDNA) (particularly for PCR) – heating at high temperature (e.g., 95 °C). The samples were heated for different periods of time resulting in a mild denaturation after a brief heating, and full DNA melting after prolonged incubation (Fig. 5C). Overall, our results indicated that BP-containing DNA may become a promising alternative for a delicate and low-cost photoimmobilization, hybridization as well as denaturation.

## Discussion

A strict temporal and spatial control provided by the UV-triggered formation of covalent junctions renders photo-induced cross-linking a powerful tool for the examination of biomolecular interactions. In addition, it is important that the UV cross-linking can be used not only for the *in vitro* studies but also for the capture of complexes *in vivo*, without perturbing their interface. One way to minimize a cross-linking heterogeneity, is a site-specific incorporation of the photoreactive functionality that readily localizes unique cross-linking contacts. Therefore, a considerable emphasis must be placed on the development of a simple as well as flexible approach for the functionalization of interacting partners. Aryl ketones (i.e., AP and BP) are very effective UV-inducible functional groups that upon irradiation generate a reactive triplet biradical, which predominantly abstracts a hydrogen atom from a donor molecule^42^. Aliphatic hydrogens or hydrogens adjacent to hydroxyl groups can serve as H-donors, depending on H steric accessibility and close proximity to AP/BP. As such, both proteins and nucleic acids, as well as a wide variety of organic and inorganic polymeric materials, may be subjected to a successful AP/BP-mediated cross-linking. Herein, we present a facile and straightforward approach to covalently couple the AP/BP-functionalized nucleic acid molecules with both proteins and diverse solid supports. We show that ssDNA oligomers 3′-tailed with AP- or BP-containing *N*^4^-modified cytidine nucleotides act as a photoprobe which is easily connected to DNA-binding proteins only upon exposure to UV light. We also observe that the efficiency of the cross-linking strongly depends on the quantity of photoactive groups present in a nucleic acid molecule. In fact, it makes *ortho-*AP/BP the least, whilst *para*-AP/BP isomers the most effective cross-linking agents. Our results highlight three important findings. First, a TdT-catalysed incorporation of dC^AP^ and dC^BP^ suggests that it is adjustable to any nucleic acid molecule regardless of its sequence context or length. Despite the presence of several alternatives for the enzymatic 3′-end labelling using other terminal transferase activity-bearing human polymerases (e.g., *θ*, *λ* or *µ*)^54–56^ or a polymerase acting as a ribozyme^57^, TdT is the only researched and marketed enzyme so far. Considering that TdT is known to extend ss- or ds- DNA^58,59^, RNA substrates^60^ as well as to perform *de novo* synthesis of polynucleotides^61^, the proposed method is of paramount importance since it can be easily adopted for the cross-linking of a wide spectrum of nucleic acids. Second, it is convenient because the number of photoreactive groups to be introduced can be easily varied. Third, irradiation at 365 nm wavelength, which can be referred to as UVA light (315–400 nm), causes absorbance by photoactive AP/BP moieties mostly, preventing direct damage and leaving native biomolecules intact.

A coupling of nanomaterials with biomolecules for the generation of functional nanosystems or nanodevices is yet another field where photo-cross-linking can emerge as a very promising approach. DNA immobilization and hybridization play a major role in the era of nanostructures, therefore all challenges and drawbacks of various immobilization procedures need to be acknowledged. Surface modification, functionalization or coating may be considered as necessary steps for the successful DNA immobilization^1,62^. Consequently, complicated surface preparation techniques affect the experimental design and limit the choice of relatively inert polymeric supports. PS, PP and BS are among the most popular biologically inert polymers, and are used in many applications. Glass substrates, in particular, are very often utilized for DNA microchips, however surface pre-treatment is necessary to attach biomolecules^63^. Our results offer an effortless way to directly immobilize any type of DNA onto a wide range of polymer substrates, including inert PS, PP and BS, by applying UV irradiation. In addition, we show that biodegradable polymers, such as PLA and PDMS, as well as the sol-gel based hybrid materials containing aluminium, vanadium, titanium or zirconium ions can be readily utilized as bioactive supports for DNA immobilization. Both PLA and silicone-based organic elastomer PDMS are widely used in targeted drug delivery and release systems, for detection of pathogens, as antisense therapeutics or as scaffolds in tissue engineering^64^. Similarly, sol-gel based composites show promise as nanocarriers, sensing devices or scaffolds for tissue repair^65^. Owing to the low cost, simple design and biocompatibility of such polymers, the functionalization will allow to expand their utilization in future.

Our findings indicate that the greater number of modified residues is present, the more efficient immobilization of such DNA molecules takes place. Remarkably, the largest 3′-tailed DNA products exhibit particularly abnormal electrophoretic mobility or no detectable mobility at all. Performing TdT-elongation in the dark did not prevent from accumulation of the material in or near the wells. Moreover, these aggregates were not digested by protease, which implied that an unknown material was not a DNA-TdT complex formed due to the inevitable exposure to UV present in a daylight. Altogether, it might be speculated that such phenomenon can be caused by the presence of a highly hydrophobic 3′-AP/BP-tail that seems likely to determine the formation of specific steric structures, aggregates or even nanoscaled particles that prevent from fractionation by conventional methods. Further, we suggest that the formation of covalent bonds between 3′-modified DNA and the solid support occurs at multiple sites rather than at a single 3′-end. Hence, a greater rigidity and stability of immobilized biomolecules is obtained which, in fact, directs DNA properly for the successful hybridization. Thermal denaturation, the most frequent and simple method of DNA denaturation, further ensures compatibility of the immobilized *N*^4^-acyl-dC-DNA probes for DNA hybridization based assays. Taken together our results show that a novel immobilization method presented here (i) does not require any prior chemical derivatization of solid supports, (ii) is suitable for a broad range of nucleic acid molecules (ss- or ds-DNA/RNA) as well as the most classic hybridization-denaturation procedures, and (iii) may prove useful for the rapid detachment of DNA from the polymeric support due to a certain pH-lability of *N*^4^-acyl-modifications.

In conclusion, a simple but effective photo-cross-linking approach based on the *N*^4^-modified-DNA bearing photoactive AP/BP modifications was designed and studied. 3′-tailed functionalized nucleic acid molecules containing several to several hundred AP or BP moieties were cross-linked with both an interacting protein and a variety of solid supports upon exposure to UV light. Successful DNA immobilization, hybridization as well as denaturation procedures were also achieved. Our findings indicate that *N*^4^-modified cytidine nucleotides bearing functional AP or BP groups can be considered as suitable building blocks for the construction of functionalized DNA for the UV-induced cross-linking.

## Methods

### Materials

All chemicals were of the highest reagent grade, unless otherwise specified. Synthetic ONs were purchased from Metabion (Planegg/Steinkirchen, Germany); sequences of the ONs are shown in Table 1. [γ-^33^P]-ATP was obtained from Perkin Elmer (Singapore). TdT, T4 polynucleotide kinase (T4 PNK), solutions of 2′-deoxyribonucleoside triphosphates, Zeba Spin desalting columns and BS glass slides were purchased from Thermo Fisher Scientific Baltics (Vilnius, Lithuania). The UV light source was purchased from Epileds (Tainan, Taiwan).

**Table 1:** Sequences of the ONs used in this study.

Oligo	Sequence
P1	5′-TAATACGACTCACTATAGGGAGA-3′
P1R	5′-TCTCCCTATAGTGAGTCGTATTA-3′
P2	5′-ATCATATGCGTCTGTGTGACCG-3

### Photo-cross-linking of 3′-dC^AP^- or 3′-dC^BP^-tailed ONs to TdT

The cross-linking apparatus was constructed as described previously, with slight modifications^66^. The apparatus consisted of an ice container, a 96-well plate, a sheet of parafilm and an UV light source. A sheet of parafilm was placed over the top of a 96-well plate, and taped to the plate on all four sides. Each well was pressed to create a shallow groove. The plate was kept on ice before and during irradiation. Samples were irradiated at 365 ± 5 nm (200–220 mW/cm^2^) 5 mm away from the surface of the light source, which provided dose of UV irradiation of ~8 J/cm^2^.

UV cross-linking of dC^AP^- or dC^BP^-elongated primer P1 to TdT was carried out after TdT-catalysed 3′-elongation reactions. The primer P1 was 5′-^33^P-labelled by using T4 PNK in the presence of [γ-^33^P]-ATP. The 5′-labelled primer was desalted using Zeba Spin desalting columns (7 K MWCO). The reaction mixtures (40 µL) consisted of TdT (0.5 U), 5′-^33^P-labelled P1 (5 nM), dCTP, dC^AP^TP or dC^BP^TP (10 µM) and glutamate reaction buffer (20 mM sodium glutamate, 20 mM NaCl, 10 mM DTT, 0.5% Triton X-100, 1 mM MgCl_2_ (pH 8.2)). The reaction mixtures were incubated for 5 min at 37 °C. Immediately after incubation, the reaction mixtures were chilled on ice and transferred as 10 µL drops to the wells on the parafilm tape. The ice container was placed underneath a 365 nm UV, and samples were irradiated for 5 min. Then the samples were transferred from parafilm wells to microtubes and quenched with 2 × loading solution. To verify the TdT-ON cross-links generated by the irradiation, the samples were supplemented with SDS loading dye, heated at 95 °C for 5 min, and analysed by electrophoresis on a 14% (w/v) SDS-PAGE gel. The cross-linking products were then visualized by phosphorimaging.

### Preparation of solid supports for photochemical immobilization

PS and PP supports were cut out of a standard sterile Petri dish and a microcentrifuge tube, respectively. PLA polymer support was received as a gift from Egidijus Šimoliūnas and was manufactured as described previously^67^. PDMS and metal-containing sol-gel composite (hybrid) supports were received as a gift from Evaldas Balčiūnas. PDMS films were prepared by mixing the silicon base (Sylgard 184, Dow Corning) with a proprietary curing agent at 10:1 mass ratio in a glass Petri dish, followed by degassing under vacuum, curing for 1 hour at 100 °C, and finally removing the film. Hybrid material films containing aluminium, titanium, zirconium or vanadium were prepared on BS slides as described previously^68–70^. All polymer supports were prepared as 20 × 7 mm slides, washed with 200 µL of ethanol and air-dried.

### Photochemical immobilization of 3′-dC^AP^- or 3′-dC^BP^-tailed ONs onto the solid supports

To immobilize dC^AP^- or dC^BP^-elongated ssDNA onto the solid supports, TdT-catalysed 3′-tailing was performed as described above, except for using a TdT buffer (supplied by the manufacturer of the enzyme). 5′-^33^P-radiolabelled ON P1 was used for the elongation and immobilization. Briefly, the reaction mixtures (150 µL) were incubated from 10 sec to 60 min at 37 °C, 10 µL samples were taken from the reaction mixture at predetermined times (10, 15, 30, 45 sec and 1, 5 and 60 min) and terminated with 1 µL of EDTA (200 mM) following a complete inactivation of TdT by heating (10 min 70 °C). After TdT inactivation, the reaction mixtures were chilled on ice and transferred as 2 µL drops onto a slide of solid support. Then the specimens were placed under the UV light source (5 mm away from the surface of the light source) and irradiated for 5 min (365 ± 5 nm). This provided dose of UV irradiation of ~8 J/cm^2^. Immediately after irradiation, the specimens were rinsed with 500 µL of wash buffer (50 mM potassium phosphate, 1% Triton X-100 (pH 7.0)) following incubation (1 h at room temperature) in 1.5 mL of wash buffer vigorously shaking. Then the specimens were rinsed with 200 µL of distilled water and air-dried. Photochemical immobilization of modified ONs to solid supports was then visualized by phosphorimaging. To determine the dependency of the UVA-induced immobilization on the length of the 3′-modified tail, the intensities of the spots representing the immobilized products were determined using OptiQuant analysis software (version 03.00, Packard Instrument Company Inc., Meriden, CT, USA).

### Hybridization of ssDNA targets to the immobilized 3′-dC^BP^-tailed complementary DNA probes

A linear ssDNA probe P1 or P2 was 3′-tailed with dC^*p*BP^ using TdT. The reaction mixture was prepared in a total volume of 10 µL and consisted of TdT (10 U), P1/P2 (500 nM), dCpBPTP (100 µM) and TdT buffer supplied by the manufacturer. The reaction mixtures were incubated for 10 min at 37 °C, quenched by the addition of 1 µL of EDTA (200 mM) following a complete inactivation of TdT by heating (10 min 70 °C). After TdT inactivation, the reaction mixtures were chilled on ice and transferred as 2.5 µL drops onto a slide of PS support. Then the specimens were placed under the UV light source (5 mm away from the surface of the light source) and irradiated for 5 min (365 ± 5 nm), which provided dose of UV irradiation of ~8 J/cm^2^. Immediately after irradiation, the specimens were rinsed with 500 µL of wash buffer (50 mM potassium phosphate, 1% Triton X-100 (pH 7.0)) following incubation (1 h at room temperature) in 1.5 mL of wash buffer vigorously shaking. Then the specimens were rinsed with 200 µL of distilled water and air-dried. Silicone was used to create a ring-fence around the area of immobilized ONs, forming a shallow groove. A primer P1R was 5′-radiolabelled as described above and diluted in a 5 × saline-sodium citrate hybridization buffer (0.75 M NaCl, 75 mM sodium-citrate, 0.02% SDS (pH 7.0)) to a final 10 nM concentration. The 5′-labelled DNA target P1R was transferred as 10 μL drops onto the immobilized DNA probes P1/P2, enclosed with silicone, and further sealed to prevent the evaporation. The specimens were incubated at room temperature, 37 °C or 55 °C, for 2 or 16 hours. The solution was removed from the wells, which were then washed six times with 1 mL of distilled water and air-dried. The DNA denaturation procedure was performed by repeatedly heating samples at 95 °C for 1, 5 or 15 min. Hybridization and denaturation results were then visualized by phosphorimaging.

## Electronic supplementary material

Supplementary information

## Data Availability

All data generated or analysed during this study are included in this published article (and its Supplementary Information files).

## References

[1] Biju V (2014). Chemical modifications and bioconjugate reactions of nanomaterials for sensing, imaging, drug delivery and therapy. Chem. Soc. Rev..

[2] Sun H, Ren J, Qu X (2016). Carbon nanomaterials and DNA: From molecular recognition to applications. Acc. Chem. Res..

[3] Ming X, Laing B (2015). Bioconjugates for targeted delivery of therapeutic oligonucleotides. Adv. Drug Deliv. Rev..

[4] Hu, Q., Li, H., Wang, L., Gu, H. & Fan, C. DNA nanotechnology-enabled drug delivery systems. *Chem. Rev*., 10.1021/acs.chemrev.7b00663 (2018). 10.1021/acs.chemrev.7b00663 29465222

[5] Hu R (2014). DNA nanoflowers for multiplexed cellular imaging and traceable targeted drug delivery. Angew. Chem..

[6] Meng HM (2016). Aptamer-integrated DNA nanostructures for biosensing, bioimaging and cancer therapy. Chem. Soc. Rev..

[7] Silvi S, Credi A (2015). Luminescent sensors based on quantum dot-molecule conjugates. Chem. Soc. Rev..

[8] Gao L (2014). Graphene oxide-DNA based sensors. Biosens. Bioelectron..

[9] Liang H (2014). Functional DNA-containing nanomaterials: cellular applications in biosensing, imaging, and targeted therapy. Acc. Chem. Res..

[10] Torabi SF, Lu Y (2014). Functional DNA nanomaterials for sensing and imaging in living cells. Curr. Opin. Biotechnol..

[11] Linko V, Ora A, Kostiainen MA (2015). DNA nanostructures as smart drug-delivery vehicles and molecular devices. Trends Biotechnol..

[12] Gerling T, Wagenbauer KF, Neuner AM, Dietz H (2015). Dynamic DNA devices and assemblies formed by shape-complementary, non-base pairing 3D components. Science.

[13] Steen H, Jensen ON (2002). Analysis of protein-nucleic acid interactions by photochemical cross-linking and mass spectrometry. Mass Spectrom. Rev..

[14] D’Onofrio J, Montesarchio D, De Napoli L, Di Fabio G (2005). An efficient and versatile solid-phase synthesis of 5′-and 3′-conjugated oligonucleotides. Org. Lett..

[15] Raindlová V, Pohl R, Hocek M (2012). Synthesis of aldehyde-linked nucleotides and DNA and their bioconjugations with lysine and peptides through reductive amination. Chem. Eur. J..

[16] Dadová J (2013). Vinylsulfonamide and acrylamide modification of DNA for cross-linking with proteins. Angew. Chem. Int. Ed..

[17] Olszewska A, Pohl R, Brázdová M, Fojta M, Hocek M (2016). Chloroacetamide-linked nucleotides and DNA for cross-linking with peptides and proteins. Bioconjugate Chem..

[18] Olszewska A, Pohl R, Hocek M (2017). Trifluoroacetophenone-linked nucleotides and DNA for studying of DNA–protein interactions by 19F NMR spectroscopy. J. Org. Chem..

[19] Flett FJ, Walton JG, Mackay CL, Interthal H (2015). Click chemistry generated model DNA-peptide heteroconjugates as tools for mass spectrometry. Anal. Chem..

[20] Yeo JE (2014). Synthesis of site-specific DNA-protein conjugates and their effects on DNA replication. ACS Chem. Biol..

[21] Horáková P (2011). Tail-labelling of DNA probes using modified deoxynucleotide triphosphates and terminal deoxynucleotidyl tranferase. Application in electrochemical DNA hybridization and protein-DNA binding assays. Org. Biomol. Chem..

[22] Hollenstein M (2013). Deoxynucleoside triphosphates bearing histamine, carboxylic acid, and hydroxyl residues – synthesis and biochemical characterization. Org. Biomol. Chem..

[23] Hollenstein M, Wojciechowski F, Leumann CJ (2012). Polymerase incorporation of pyrene-nucleoside triphosphates. Bioorg. Med. Chem. Lett..

[24] Kobayashi T, Takezawa Y, Sakamoto A, Shionoya M (2016). Enzymatic synthesis of ligand-bearing DNAs for metal-mediated base pairing utilising a template-independent polymerase. ChemComm..

[25] Jarchow-Choy SK, Krueger AT, Liu H, Gao J, Kool ET (2010). Fluorescent xDNA nucleotides as efficient substrates for a template-independent polymerase. Nucleic Acids Res..

[26] Winz ML, Linder EC, André T, Becker J, Jäschke A (2015). Nucleotidyl transferase assisted DNA labelling with different click chemistries. Nucleic Acids Res..

[27] Jang EK, Ki MR, Pack SP (2017). Design of reactive-end DNA oligomers via incorporation of oxanine into oligonucleotides using terminal deoxynucleotidyl transferase. Process Biochem..

[28] Takahara M, Wakabayashi R, Minamihata K, Goto M, Kamiya N (2017). Primary amine-clustered DNA aptamer for DNA-protein conjugation catalyzed by microbial transglutaminase. Bioconjugate Chem..

[29] Röthlisberger P (2017). Facile immobilization of DNA using an enzymatic his-tag mimic. Chem Comm..

[30] Preston GW, Wilson AJ (2013). Photo-induced covalent cross-linking for the analysis of biomolecular interactions. Chem. Soc. Rev..

[31] Murale DP, Hong SC, Haque MM, Lee JS (2017). Photo-affinity labeling (PAL) in chemical proteomics: a handy tool to investigate protein-protein interactions (PPIs). Proteome Sci..

[32] Qiu Z, Lu L, Jian X, He C (2008). A diazirine-based nucleoside analogue for efficient DNA interstrand photocross-linking. J. Am. Chem. Soc..

[33] Winnacker M, Breeger S, Strasser R, Carell T (2009). Novel diazirine-containing DNA photoaffinity probes for the investigation of DNA-protein-interactions. Chem Bio Chem..

[34] Lercher L, McGouran JF, Kessler BM, Schofield CJ, Davis BG (2013). DNA modification under mild conditions by Suzuki-Miyaura cross-coupling for the generation of functional probes. Angew. Chem. Int. Ed..

[35] Shigdel UK, Zhang J, He C (2008). Diazirine-based DNA photo-cross-linking probes for the study of protein-DNA Interactions. Angew. Chem. Int. Ed..

[36] Smith CC, Hollenstein M, Leumann CJ (2014). The synthesis and application of a diazirine-modified uridine analogue for investigating RNA-protein interactions. RSC Adv..

[37] Chin JW, Martin AB, King DS, Wang L, Schultz PG (2002). Addition of a photocrosslinking amino acid to the genetic code of *Escherichia coli*. Proc. Natl. Acad. Sci..

[38] Ai HW, Shen W, Sagi A, Chen PR, Schultz PG (2011). Probing protein-protein interactions with a genetically encoded photo-crosslinking amino acid. Chembiochem.

[39] Ito Y (2006). Photoimmobilization for microarrays. Biotechnol. Prog..

[40] Dankbar DM, Gauglitz G (2006). A study on photolinkers used for biomolecule attachment to polymer surfaces. Anal. Bioanal. Chem..

[41] Arndt S, Wagenknecht HA (2014). “Photoclick” postsynthetic modification of DNA. Angew. Chem. Int. Ed..

[42] Dormán G, Nakamura H, Pulsipher A, Prestwich GD (2016). The life of Pi star: exploring the exciting and forbidden worlds of the benzophenone photophore. Chem. Rev..

[43] Martin TA (2011). Quantitative photochemical immobilization of biomolecules on planar and corrugated substrates: a versatile strategy for creating functional biointerfaces. ACS Appl. Mater. Interfaces.

[44] Martinez JS, Lehaf AM, Schlenoff JB, Keller TC (2013). Cell durotaxis on polyelectrolyte multilayers with photogenerated gradients of modulus. Biomacromolecules.

[45] Dhende VP, Samanta S, Jones DM, Hardin IR, Locklin J (2011). One-step photochemical synthesis of permanent, nonleaching, ultrathin antimicrobial coatings for textiles and plastics. ACS Appl. Mater. Interfaces.

[46] Loschonsky S (2008). Surface-attached PDMAA-GRGDSP hybrid polymer monolayers that promote the adhesion of living cells. Biomacromolecules.

[47] Martin TA, Caliari SR, Williford PD, Harley BA, Bailey RC (2011). The generation of biomolecular patterns in highly porous collagen-GAG scaffolds using direct photolithography. Biomaterials.

[48] Renberg B (2009). Serial DNA immobilization in micro-and extended nanospace channels. Lab Chip.

[49] Le Goff GC, Blum LJ, Marquette CA (2013). Shrinking hydrogel-DNA spots generates 3D microdots arrays. Macromol. Biosci..

[50] Kauer JC, Erickson-Viitanen S, Wolfe R, DeGrado WF (1986). p-Benzoyl-L-phenylalanine, a new photoreactive amino acid. Photolabeling of calmodulin with a synthetic calmodulin-binding peptide. J. Biol. Chem..

[51] Lee HS, Dimla RD, Schultz PG (2009). Protein-DNA photo-crosslinking with a genetically encoded benzophenone-containing amino acid. Bioorg. Med. Chem. Lett..

[52] Larsen EKU, Mikkelsen MBL, Larsen NB (2014). Facile photoimmobilization of proteins onto low-binding PEG-coated polymer surfaces. Biomacromolecules.

[53] Jakubovska Jevgenija, Tauraitė Daiva, Birštonas Lukas, Meškys Rolandas (2018). N 4-acyl-2′-deoxycytidine-5′-triphosphates for the enzymatic synthesis of modified DNA. Nucleic Acids Research.

[54] Kent T, Mateos-Gomez PA, Sfeir A, Pomerantz RT (2016). Polymerase *θ* is a robust terminal transferase that oscillates between three different mechanisms during end-joining. eLife.

[55] Ramadan K (2003). Human DNA polymerase *λ* possesses terminal deoxyribonucleotidyl transferase activity and can elongate RNA primers: implications for novel functions. J. Mol. Biol..

[56] Andrade P, Martín MJ, Juárez R, de Saro FL, Blanco L (2009). Limited terminal transferase in human DNA polymerase *μ* defines the required balance between accuracy and efficiency in NHEJ. Proc. Natl. Acad. Sci..

[57] Samanta Biswajit, Horning David P, Joyce Gerald F (2018). 3′-End labeling of nucleic acids by a polymerase ribozyme. Nucleic Acids Research.

[58] Motea EA, Berdis AJ (2010). Terminal deoxynucleotidyl transferase: the story of a misguided DNA polymerase. Biochim. Biophys. Acta, Proteins Proteomics.

[59] Roychoudhury R, Jay E, Wu R (1976). Terminal labeling and addition of homopolymer tracts to duplex DNA fragments by terminal deoxynucleotidyl transferase. Nucleic Acids Res..

[60] Zhao B, Gong Z, Ma Z, Wang D, Jin Y (2011). Simple and sensitive microRNA labeling by terminal deoxynucleotidyl transferase. Acta Biochim. Biophys. Sin..

[61] Ramadan K, Shevelev IV, Maga G, Hübscher U (2004). *De novo* DNA synthesis by human DNA polymerase λ, DNA polymerase μ and terminal deoxyribonucleotidyl transferase. J. Mol. Biol..

[62] Rashid JI, Yusof NA (2017). The strategies of DNA immobilization and hybridization detection mechanism in the construction of electrochemical DNA sensor: a review. Sens. Biosens. Res..

[63] Vong T (2009). Site-specific immobilization of DNA in glass microchannels via photolithography. Langmuir.

[64] Maitz MF (2015). Applications of synthetic polymers in clinical medicine. Biosurf. Biotribol..

[65] Owens GJ (2016). Sol–gel based materials for biomedical applications. Progr. Mater. Sci..

[66] Sontheimer EJ (1994). Site-specific RNA crosslinking with 4-thiouridine. Mol. Biol. Rep..

[67] Malinauskas M (2014). 3D microporous scaffolds manufactured via combination of fused filament fabrication and direct laser writing ablation. Micromachines.

[68] Sakellari I (2010). Two-photon polymerization of titanium-containing sol-gel composites for three-dimensional structure fabrication. Appl. Phys. A Mater. Sci. Process..

[69] Ovsianikov A (2008). Ultra-low shrinkage hybrid photosensitive material for two-photon polymerization microfabrication. ACS Nano..

[70] Kabouraki E (2013). Redox multiphoton polymerization for 3D nanofabrication. Nano Lett..

