# Supplementary material for: A versatile method for the UVA-induced cross-linking of acetophenone- or benzophenone-functionalized DNA
Source: Sci Rep. 2018 Nov 7;8:16484. doi: 10.1038/s41598-018-34892-9 (PMC6220319; doi:10.1038/s41598-018-34892-9)
Supplement: Supplementary file 1 — Supplementary information [file 41598_2018_34892_MOESM1_ESM.pdf]

Supplementary information

A versatile method for the UVA-induced cross-linking of acetophenone- or benzophenone-functionalized DNA

Jevgenija Jakubovska*, Daiva Tauraitė and Rolandas Meškys

Department of Molecular Microbiology and Biotechnology, Institute of Biochemistry, Life Sciences Center, Vilnius University, Sauletekio al. 7, LT-10257 Vilnius, Lithuania

*Corresponding Author: e-mail: jevgenija.jakubovska@bchi.vu.lt.

CONTENTS:

Native PAGE showing cross-linking of modified DNA with SSB and BSA….p.S2

Agarose gel electrophoresis of long 3'-dC^{pBP}-tailed ONs………….……….p.S3

Pulsed-field gel electrophoresis of long 3'-dC^{pBP}-tailed ONs………………...p.S4

Experimental procedures……………………………………………………….p.S5

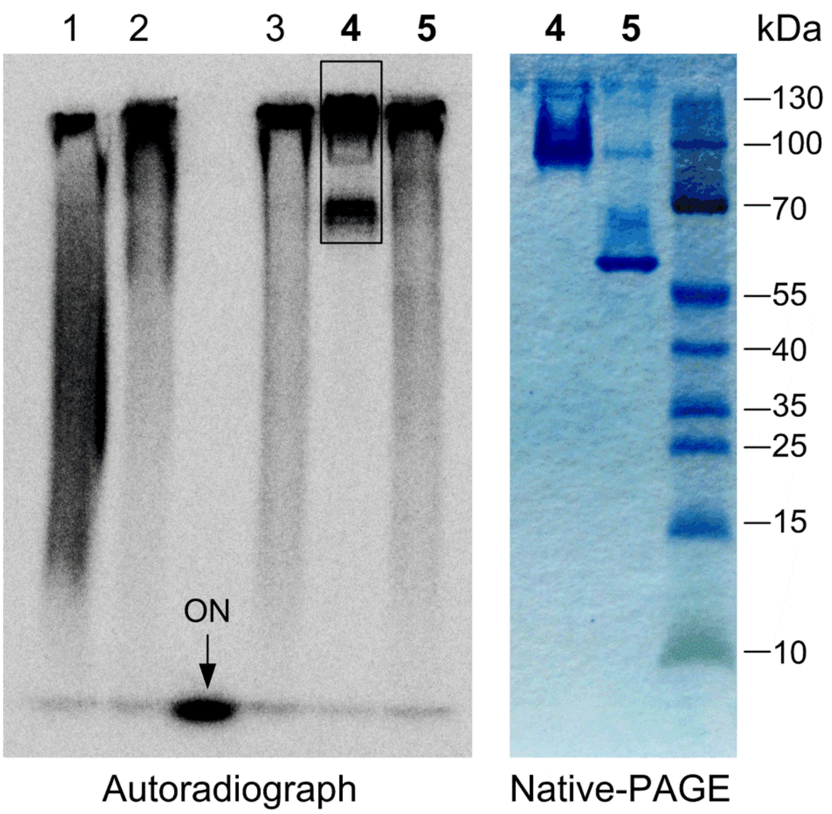

Figure S1. An autoradiogram of the native polyacrylamide gel (left) and Coomassie staining of the same gel (right) showing UVA-induced cross-linking of dC^{pBP}-containing DNA to proteins. Lane 1, dC^{pBP}-ON:TdT UV-free control; lane 2, UV cross-linked complexes of dC^{pBP}-ON:TdT; lane 3, UV exposed control sample after removing TdT; lane 4, UV cross-linked complexes of dC^{pBP}-ON:SSB; lane 5, UV exposed dC^{pBP}-ON:BSA control. Molecular mass marker (kDa) – PageRuler prestained protein ladder (Thermo Scientific).

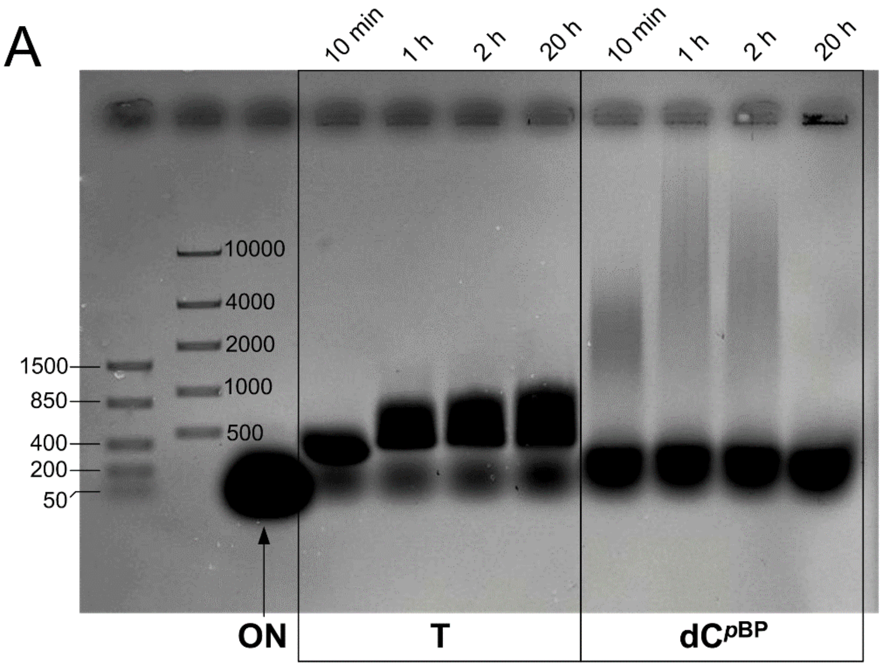

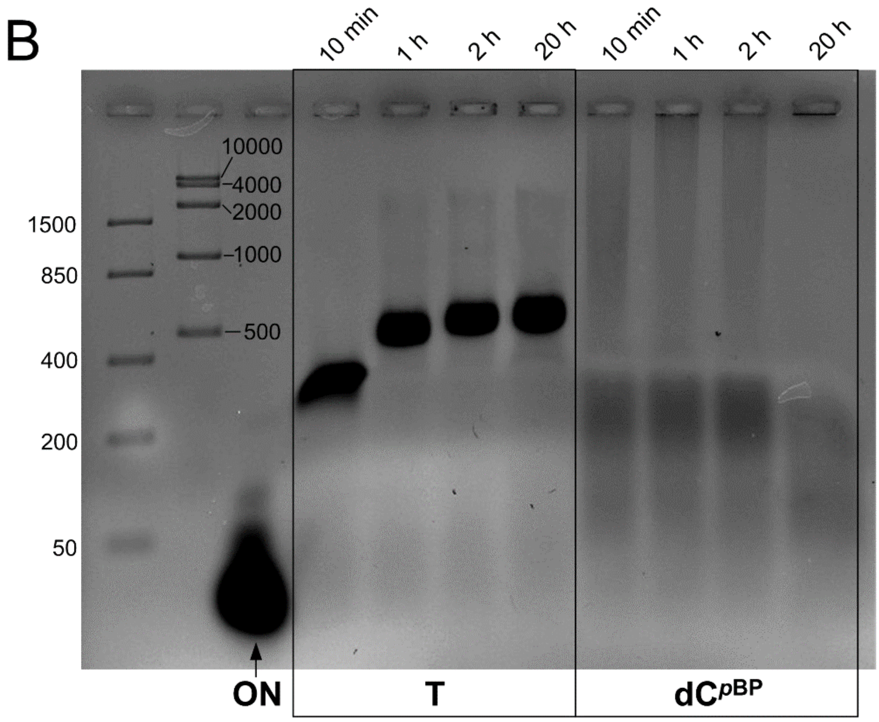

Figure S2. Fluorescence images of agarose gels showing electrophoretic migration of a 3'-elongated ON using TdT and TTP or dC^{pBP}TP. (**A**) 0.8 % agarose gel, TBE buffer; (**B**) 2.5 % agarose gel, TAE buffer. The elongation time and used dNTPs are indicated above and below the lanes, respectively. DNA ladders used (kb): FastRuler DNA ladder, low range (Thermo Scientific) (left); FastRuler DNA ladder, high range (Thermo Scientific) (right).

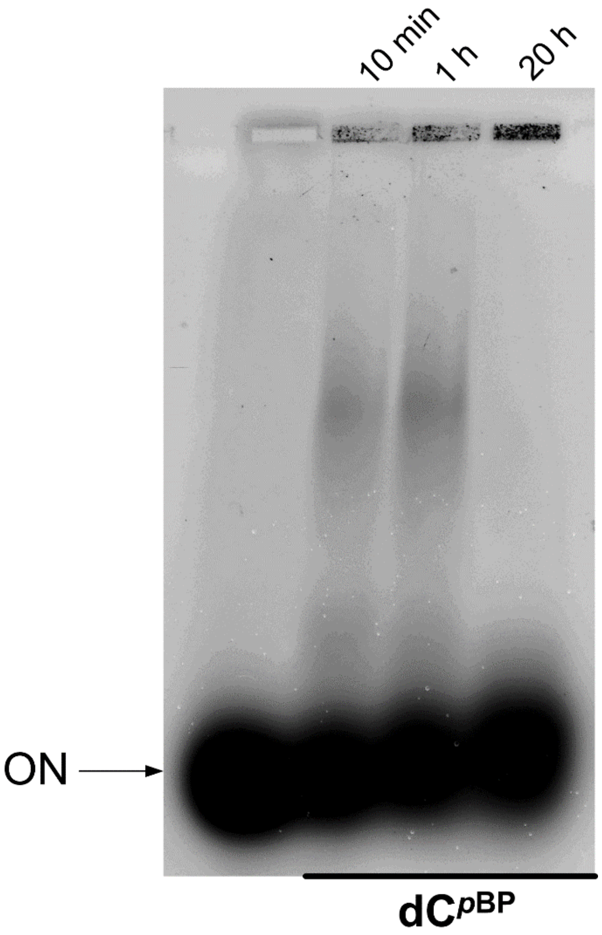

Figure S3. A fluorescence image of a pulsed-field agarose gel showing electrophoretic migration of a 3'-elongated ON using TdT and dC^{pBP}TP. The elongation time is indicated above the lanes.

Photo-cross-linking of 3'-dC^{BP}-tailed ONs to SSB and BSA

The cross-linking apparatus was constructed as described previously, with slight modifications[66]. The apparatus consisted of an ice container, a 96-well plate, a sheet of parafilm and an UV light source. A sheet of parafilm was placed over the top of a 96-well plate, and taped to the plate on all four sides. Each well was pressed to create a shallow groove. The plate was kept on ice before and during irradiation. Samples were irradiated at 365±5 nm (200–220 mW/cm^2) 5 mm away from the surface of the light source, which provided dose of UV irradiation of ~8 J/cm^2.

UV cross-linking of dC^{BP}-elongated primer **P1** to SSB/BSA was carried out after TdT-catalysed 3'-elongation reactions. The primer **P1** was 5'-^{33}P-labelled by using T4 PNK in the presence of [γ-^{33}P]-ATP. The 5'-labelled primer was desalted using Zeba Spin desalting columns (7K MWCO). The reaction mixtures (20 µL) consisted of TdT (0.5 U), 5'-^{33}P-labelled **P1** (5 nM), dC^{pBP}TP (10 µM) and glutamate reaction buffer (20 mM sodium glutamate, 20 mM NaCl, 10 mM DTT, 0.5 % Triton X-100, 1 mM $MgCl_2$ (pH 8.2)). The reaction mixtures were incubated for 5 min at 37 °C. Immediately after incubation, the reaction mixtures were heated at 95 °C for 5 min. Then Proteinase K (20 µg) (Thermo Scientific) was added and the mixtures were incubated for 60 min at 65 °C. Proteinase K was inhibited using phenylmethylsulfonyl fluoride (PMSF) (1 mM, incubation for 30 min at 4 °C). Then the samples were supplemented with either SSB (10 µg) (Thermo Scientific) or BSA (1 µg) (Thermo Scientific) and incubated for 30 min at room temperature. After incubation reaction mixtures were transferred as 10 µL drops to the wells on the parafilm tape. The ice container was placed underneath a 365 nm UV, and samples were irradiated for 5 min. To verify the protein-ON cross-links generated by the irradiation, the samples were supplemented with native PAGE sample buffer and analysed by electrophoresis on a 14 % (w/v) native PAGE gel. The cross-linking products were then visualized by phosphorimaging, proteins were stained with Coomasie Briliant Blue staining solution (Applichem, Darmstadt, Germany).

Agarose gel electrophoresis of long 3'-dC^{pBP}-tailed ONs

The primer P1 bearing a 6-carboxyfluorescein at the 5'-end (50 pmol) was elongated with TdT (20 U) using dC^{pBP}TP (1 mM) in a buffer supplied by the manufacturer for TdT. The reaction mixtures were incubated at 37 °C for 10 min, 1 h or 20 h, following TdT inactivation by heating for 10 min at 70 °C. Then the samples were supplemented with a 6 × DNA gel loading dye and analysed by a conventional electrophoresis on a 0.8 % or 2.5 % agarose gels using TBE or TAE buffers, respectively. Alternatively, the samples were mixed with 1.2 % low-melting agarose and analysed by pulsed-field gel electrophoresis (1.2 % agarose gel). Pulsed-field gel electrophoresis was performed for 5 h at 6 V/cm and 8°C with a pulse time of 15 (E/W) to 15 s (S/N) using the Gene Navigator system from Pharmacia Biotech. The gels were stained for 15 min with ethidium bromide (0.5 µg/ml). Visualization was performed by fluorescence imaging using FLA-5100 imaging system (FUJIFILM, Tokyo, Japan).
